# High Milk-Clotting Activity Expressed by the Newly Isolated *Paenibacillus* spp. Strain BD3526

**DOI:** 10.3390/molecules21010073

**Published:** 2016-01-12

**Authors:** Feng Hang, Peiyi Liu, Qinbo Wang, Jin Han, Zhengjun Wu, Caixia Gao, Zhenmin Liu, Hao Zhang, Wei Chen

**Affiliations:** 1 State Key Laboratory of Food Science and Technology, School of Food Science and Technology, Jiangnan University, Wuxi 214122, China; fhang0427@126.com (F.H.); zhanghao@jiangnan.edu.cn (H.Z.); 2 State Key Laboratory of Dairy Biotechnology, Technology Center and Dairy Research Institute of Bright Dairy & Food Co. Ltd., Shanghai 200436, China; liupeiyi@brightdairy.com (P.L.); wangqinbo@brightdairy.com (Q.W.); hanjin@brightdairy.com (J.H.); wuzhengjun@brightdairy.com (Z.W.); gaocaixia@brightdairy.com (C.G.); liuzhenmin@brightdairy.com (Z.L.); 3 Synergetic Innovation Center of Food Safety and Nutrition, Jiangnan University, Wuxi 214122, China; 4 Beijing Innovation Centre of Food Nutrition and Human Health, Beijing Technology & Business University, Beijing 100048, China

**Keywords:** *Paenibacillus*, coagulant, milk clotting activity, proteolytic activity

## Abstract

*Paenibacillus* spp. BD3526, a bacterium exhibiting a protein hydrolysis circle surrounded with an obvious precipitation zone on skim milk agar, was isolated from raw yak (*Bos grunniens*) milk collected in Tibet, China. Phylogenetic analysis based on 16S rRNA and whole genome sequence comparison indicated the isolate belong to the genus *Paenibacillus*. The strain BD3526 demonstrated strong ability to produce protease with milk clotting activity (MCA) in wheat bran broth. The protease with MCA was predominantly accumulated during the late-exponential phase of growth. The proteolytic activity (PA) of the BD3526 protease was 1.33-fold higher than that of the commercial *R. miehei* coagulant. A maximum MCA (6470 ± 281 SU mL^−1^) of the strain BD3526 was reached under optimal cultivation conditions. The protease with MCA was precipitated from the cultivated supernatant of wheat bran broth with ammonium sulfate and purified by anion-exchange chromatography. The molecular weight of the protease with MCA was determined as 35 kDa by sodium dodecyl sulfate-polyacrylamide gels electrophoresis (SDS-PAGE) and gelatin zymography. The cleavage site of the BD3526 protease with MCA in κ-casein was located at the Met_106_–Ala_107_ bond, as determined by mass spectrometry analysis.

## 1. Introduction

Traditionally used as a milk coagulant, calf rennet plays a critical role in the production of cheese. Chymosin (EC3.4.23.4), a predominant aspartic protease (AP) in calf rennet, specifically cleaves the Phe_105_–Met_106_ peptide bond in the κ-casein (κ-CN). After cleaving κ-CN, the destabilized casein micelles consequently aggregate to form cheese curd. However, decrease in the global supply of calf rennet *versus* the increasing demand of coagulant in the production of cheese necessitates the exploration for potential substitutes [1]. Candidate calf rennet substitutes has mainly been resourced from animals beyond calf [2,3,4], plants [5,6,7,8,9,10,11,12], genetic engineering and microorganisms [13].

The extracellular proteases of many microorganisms behave similarly to chymosin, and are therefore potential alternatives for rennet. Proteases from over 100 fungal sources have been reported to display milk-clotting activity (MCA) and fungal coagulants, especially those originated from *Rhizomucor miehei*, *R. pusillus* and *Endothia parasitica*, have already been widely used in commercial cheese-making [14]. In contrast, few commercial applications of bacterial coagulants have been reported, despite a marked increase in the documentation of bacterial proteases exerting MCA [15,16,17,18]. Recently, the milk-clotting proteases from *Bacillus amyloliquefaciens* and *Bacillus* spp. P45 have been successfully applied to the preparation of cheddar and cream cheese [19,20]. So far, there have been few reports on milk-clotting enzymes expressed by members of the genus *Paenibacillus*.

In this study, a potentially new coagulant producer, *Paenibacillus* spp. BD3526, was screened and identified. The parameters of the BD3526 protease with MCA in milk curding was assessed and compared with some commercial coagulants. The cultivation conditions were optimized for the strain BD3526 to express maximum MCA. The BD3526 protease with MCA was precipitated from the cultivated supernatant of wheat bran broth with 60% saturated ammonium sulfate and purified by anion-exchange chromatography. The molecular weight of the protease was determined by SDS-PAGE and gelatin zymography. The cleavage site of the BD3526 protease in κ-CN was determined by mass spectrometry analysis.

## 2. Results and Discussions

### 2.1. Morphological Characteristics of BD3526

**Figure 1:**
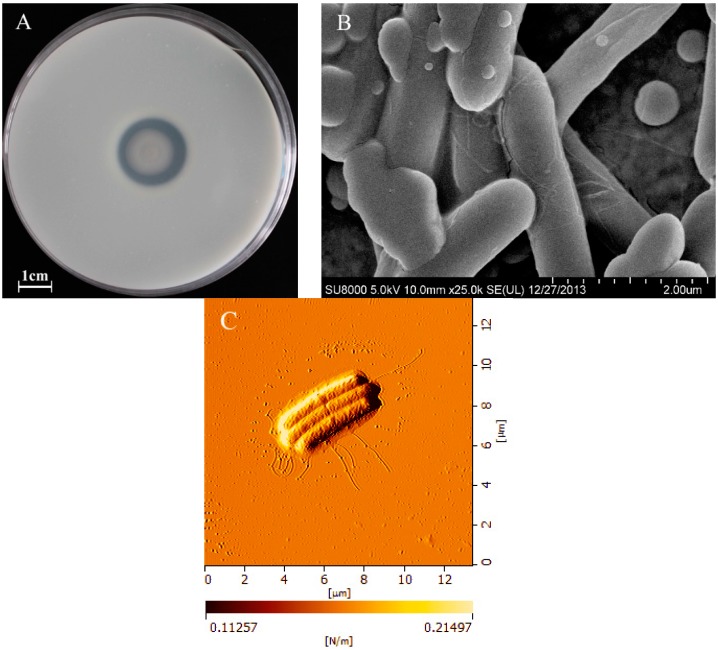
The protein hydrolysis zone and casein precipitation circle of a single colony of *Paenibacillus* spp. BD3526 on skim milk agar (**A**) and the micrographic characteristics of cell observed by scanning electron microscopy (**B**) and atomic force microscope (**C**).

The BD3526 colony exhibited an obvious protein hydrolysis circle around with a casein precipitation zone on skim milk agar (Figure 1A), indicating that the strain secretes protease with promising MCA [17]. 

The BD3526 cells were Gram-positive, motile, end-rounded and non-swelling rods with peritrichous flagella (Figure 1C). The cells were 0.4~0.6 μm in width and 5~8 μm in length (Figure 1B). The BD3526 strain was proposed as a novel species belonging to the genus *Paenibacillus*, based on the results of 16S rRNA and whole genome sequencing. The 16S rRNA and whole genome sequence of this strain is available in GenBank under the accession No. KM978955.1 and No. CP013023.1, respectively. Based on 16S rRNA and whole genome sequence comparison, although the strain BD3526 is most closely related to the recently described *P. shenyangensis* A9^T^ (= JCM 19307^T^ = CGMCC 2040^T^), and a homologous gene sequence possible for encoding the protease in the latter has been submitted, no assay of the enzyme has been carried out [21].

### 2.2. Effect of Concentration of Wheat Bran on the Expression of MCA by BD3526

**Figure 2:**
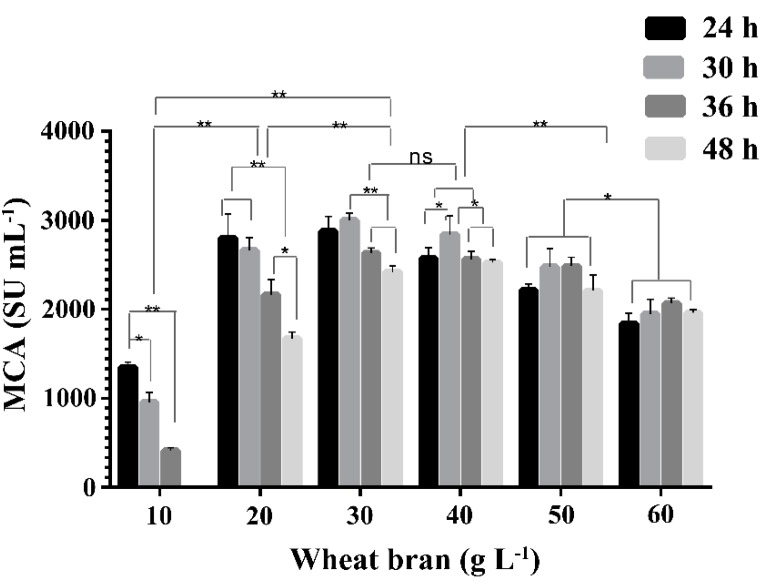
The milk-clotting activity (MCA) in the BD3526 cultivated supernatants of wheat bran broth of different concentrations and periods. The cultivation was carried out in 250 mL flask containing 50 mL wheat bran broth at 30 °C and 180 rpm. The columns were shown as mean ± S.D. *: *p* < 0.05, **: *p* < 0.01 and ns: no significant (*p >* 0.05).

Wheat bran has been reported as an ideal protease production medium for microorganisms [22,23]. The effect of wheat bran broth with concentrations from 1% to 6% (*w*/*v*) on the expression of MCA by BD3526 at 30 °C and 180 rpm was investigated. Wheat bran concentration and cultivation time had a significant influence on the protease production by BD3526 (*p* < 0.01, Figure 2). The MCA in 1% and 2% (*w*/*v*) wheat bran broth showed a downward trend at all points observed, whereas those in 3%–6% (*w*/*v*) wheat bran broth tended to rise first and then fall gradually. In 1% (*w*/*v*) wheat bran broth, no residual MCA was observed at 48 h, probably due to depletion of nutrients [24] and reduced viable cell density. The optimal concentration of wheat bran for protease production by BD3526 was 30 g·L^−1^ and peak MCA was achieved at 30 h of cultivation. At higher concentrations, the wheat bran broths were much thicker, which might hinder oxygen dissolving, and thus limit the propagation of the BD3526 cells, leading to low expression of protease. Although the optimal concentration of wheat bran broth required for BD3526 to produce protease was much lower than that for *B. amyloliquefaciens* D4 (180 g·L^−1^), the peak MCA observed in the former was much higher [16].

Microbial proteases are largely accumulated during post-exponential and stationary phases, and thus are generally regulated by carbon and nitrogen stress [25]. The protease with MCA was accumulated in the late-exponential phase of BD3526 (12–18 h). The cultivation time required to reach the peak MCA by BD3526 was longer than those required by *Enterococcus faecalis* TUA2495L [26], *B. subtilis* YB-3 [27] and *B. subtilis* natto [28], but much shorter than those required by *B. amyloliquefaciens* JNU002 [17], *B. subtilis* [22], *B. licheniformis* USC13 [29], and fungal milk-clotting protease producers [24,30,31,32,33]. The rapid production of the enzyme by BD3526 seems to be a promising advantage for industrial purposes.

### 2.3. Effect of Rotation Speeds on the Expression of MCA by BD3526

**Figure 3:**
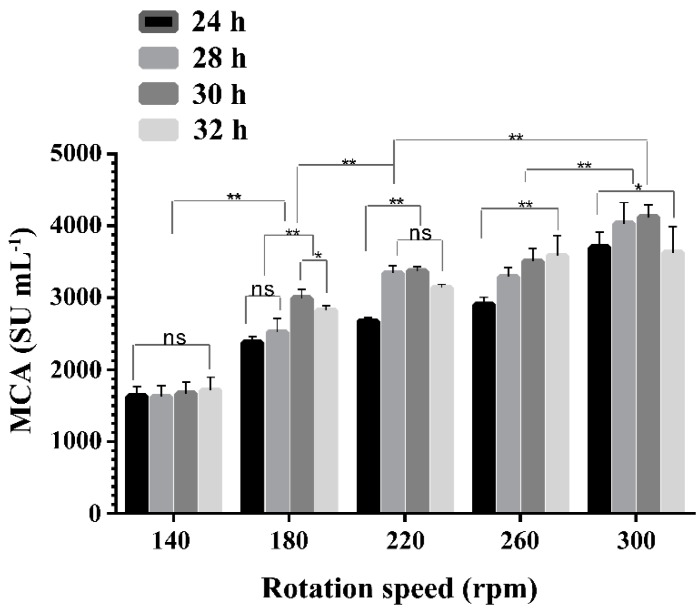
The milk-clotting activity (MCA) in the BD3526 cultivated supernatants of wheat bran broth at different rotation speeds. The cultivation was carried out in 250 mL flask containing 50 mL wheat bran broth at 30 °C. The columns were shown as mean ± S.D. *: *p* < 0.05, **: *p* < 0.01 and ns: no significant (*p >* 0.05).

Rotation speeds had significant effects on the production of protease with MCA by BD3526 (*p* < 0.01). The MCA in the supernatants of 3% (*w*/*v*) wheat bran broth at different rotation speeds and cultivation times were shown in Figure 3. Increasing rotation speeds from 140 to 300 rpm were beneficial for BD 3526 to express protease, resembling the results obtained in *R. miehei* [34], which indicated oxygen supply should be crucial for enzyme production by BD3526 in liquid cultures. The highest MCA was observed at 300 rpm (4120 ± 174 SU mL^−1^), the upper limit of the rotary shaker used in the study (Yiheng HZQ-X300C, Shanghai, China). It could be speculated that at higher rotation speeds or enhanced levels of oxygen dissolution, even higher levels of MCA might be achieved. The optimal rotation speed of 300 rpm for the BD3526 strain to produce protease was higher than those required for *B. amyloliquefaciens* D4 [35], *M. mucedo* DSM 809 [33] and *B. subtilis* [22] in shake-flask fermentations, which might be caused by the difference in the medium or the rotating semi-diameter of the shaker employed.

### 2.4. Effect of Liquid Volume on the Expression of MCA by BD3526

**Figure 4:**
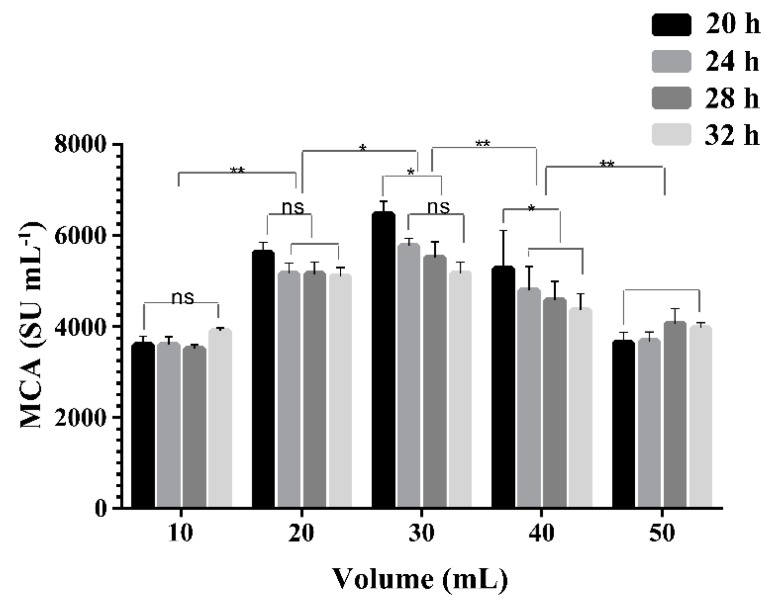
Effects of the liquid volume in flask on milk-clotting activity (MCA) expressed by the *Paenibacillus* spp. BD3526. The cultivation was carried out in 250 mL flask at 30 °C and 300 rpm. The columns were shown as mean ± S.D. *: *p* < 0.05, **: *p* < 0.01 and ns: no significant (*p >* 0.05).

The oxygen transfer coefficient K_La_ in shake-flasks has been reported to decrease with the increment in liquid volume in the flask and increase with the enhancement of the shaker speeds [36]. To investigate the concentration of dissolved oxygen in the medium on the MCA expression, the liquid volume in the flask was reduced from 50 mL to 10 mL and the cultivation in 3% (*w*/*v*) wheat bran broth was carried out at 30 °C and 300 rpm. The MCA in the supernatant increased as the volume of liquid decreased from 50 to 30 mL, but decreased thereafter (Figure 4). The viable cell counts were 7.5 × 10^8^, 8.1 × 10^8^, 1.2 × 10^9^, 7.8 × 10^8^ and 7.3 × 10^8^ CFU mL^−1^ in 10, 20, 30, 40 and 50 mL of medium in 250 mL flasks respectively, after 20 h cultivation. Higher availability of dissolved oxygen favored a high cell density of BD3526 and ultimately resulted in a high expression of MCA. The maximum MCA of 6470 ± 281 SU mL^−1^ was achieved in 30 mL broth in 250 mL flask at 20 h and 300 rpm, much higher than the peak MCA observed in 50 mL liquid in 250 mL flask at 300 rpm for 30 h (Figure 3). Our result was in agreement with that reported by Ding [18], who found the peak MCA was enhanced 1.33-fold and cultivation time was shortened by 18 h through a two-stage oxygen supply control strategy. The MCA expressed by BD3526 was much higher than most reported microorganisms in submerged fermentation (Table 1) [22,33,37,38,39,40,41,42].

**Table 1:** Comparison of milk-clotting protease production, type and molecular weight among different microorganisms.

Microorganism	Enzyme Production	Type	MW (kDa)	Reference
*R. miehei* NRRL 3420	1200 SU mL^−1^	AP	-	[38]
*R. pusillus*	1026 SU mg^−1^ protein	AP	49	[43]
*M.mucedo* DSM 809	130 SU mL^−1^	AP	32.7	[33,44]
*Thermomucor indicae-seudaticae* N31	60.5 SU mL^−1^	AP	-	[23]
*Amylomyces rouxii*	1.9 SU mL^−1^	AP	40	[45,46]
*B. amyloliquefaciens* D4	2683 SU mL^−1^	MP	58.2	[16,35]
*B. amyloliquefaciens* JNU002	6590.41 SU mL^−1^	MP	28	[17,18]
*B. subtilis* natto	685.7 SU mL^−1^	-	-	[28]
*B. subtilis* B1	1129.05 SU mL^−1^	-	-	[15]
*B. subtilis* YB-3	200 SU mL^−1^	MP	42	[27]
*B. subtilis* MTCC 10422	-	-	27	[47]
*B. sphaericus* NRC 24	1212 SU mL^−1^	SP	-	[39]
*B. licheniformis* USC13	45 U mL^−1^ *	SP	34	[29]

* Determination and definition of milk-clotting activity were different from Soxhlet unite; AP, aspartic protease; MP, metalloprotease; SP, serine protease; MW, molecular weight; -, not mentioned.

### 2.5. Effect of Cultivation Temperature on the Expression of MCA by BD3526

**Figure 5:**
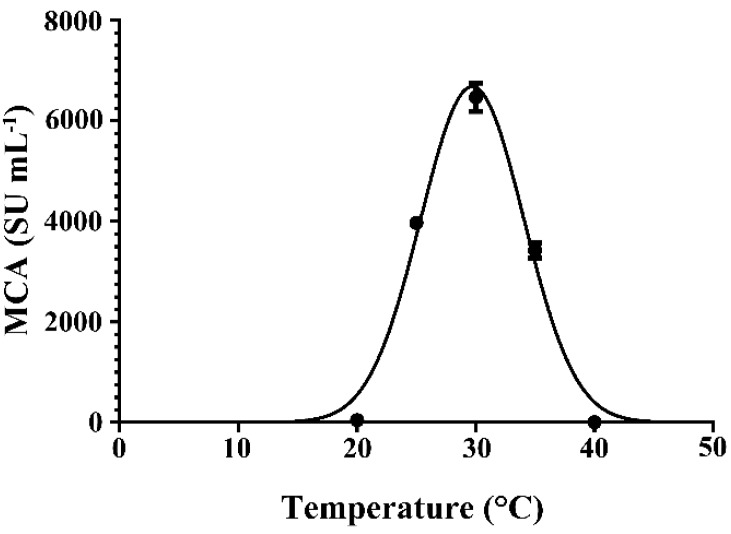
Effects of the cultivation temperature on milk-clotting activity (MCA) expressed by the *Paenibacillus* spp. BD3526. The cultivation was carried out in 250 mL flask containing 30 mL wheat bran broth at 300 rpm. Each point standards the average value from three replicates.

The production of enzymes by microorganisms is profoundly affected by cultivation temperature. The effect of cultivation temperature on the expression of MCA by BD3526 in 3% (*w*/*v*) wheat bran broth at 300 rpm for 20 h was shown in Figure 5. The MCA peaked at 30 °C, close to the optimal temperature required by *Amylomyces rouxii* [45], *Aspergillus oryzae* [48], *Mucor* spp. J20 [30] to express coagulants, but lower than those required by *B. amyloliquefaciens* D4 [35], *B. subtilis* natto [28] and *Mucor miehei* [49,50]. At cultivation temperatures higher or lower than 30 °C, the MCA expressed by BD3526 decreased significantly (*p* < 0.01). The MCA was almost undetectable when the strain was cultivated at temperatures lower than 20 °C or exceeding 40 °C.

### 2.6. Characterization of BD3526 Protease

**Figure 6:**
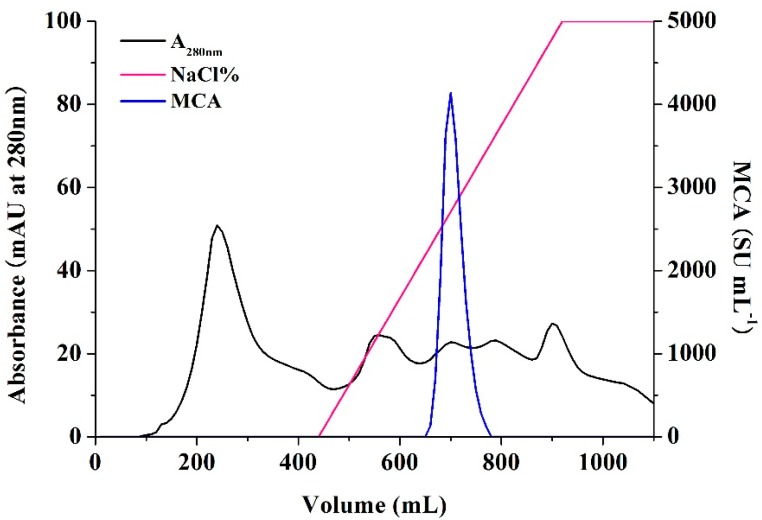
The elution profile of the crude *Paenibacillus* spp. BD3526 milk-clotting protease on the DEAE Sepharose Fast Flow chromatography column.

**Figure 7:**
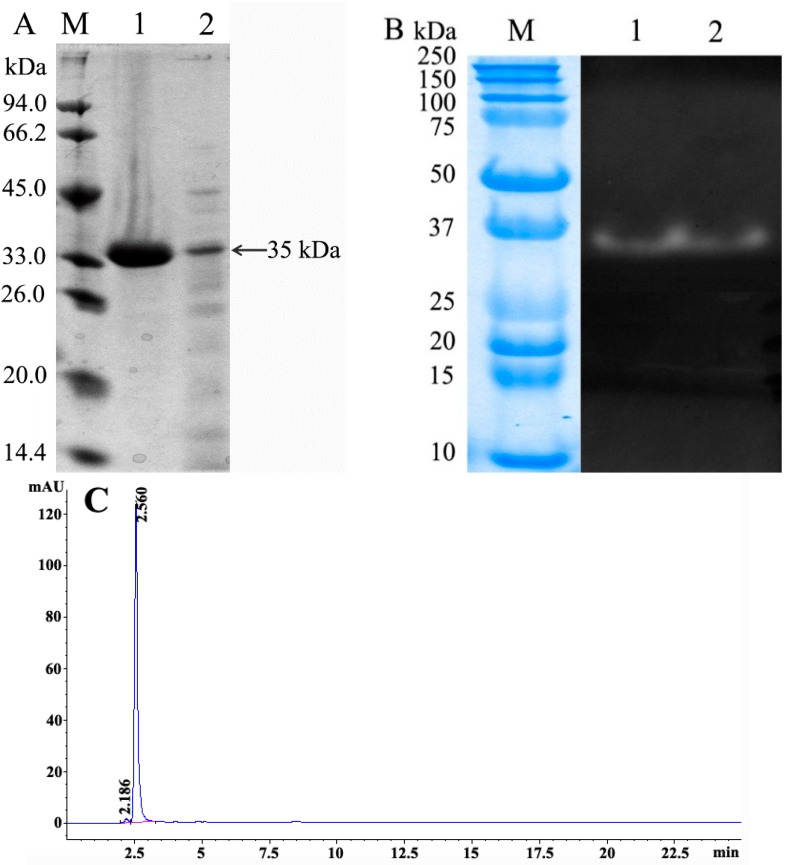
(**A**) Sodium dodecyl sulfate polyacrylamide gels electrophoresis patterns of crude and purified *Paenibacillus* spp. BD 3526 milk-clotting protease. Lane M, molecular weight marker proteins; lane 1, SDS-PAGE profile of the purified enzyme; lane 2, SDS-PAGE profile of the crude enzyme; (**B**) Gelatin zymography of purified and crude *Paenibacillus* spp. BD 3526 milk-clotting protease. Lane 1, gelatin zymography of the purified enzyme and Lane 2, gelatin zymography of the crude enzyme; (**C**) The hydrophilic interaction liquid chromatography (HILIC) elution profile of the purified *Paenibacillus* spp. BD 3526 milk-clotting protease.

The crude milk-clotting proteases were separated into five peaks on the DEAE Sepharose Fast Flow column eluted with a linear gradient of NaCl from 0 to 0.75 M, with only one peak exerting MCA (Figure 6). The targeted protein was eluted out at 0.38–0.45 M NaCl. The purified enzyme was detected to be a single band with an apparent molecular mass of 35 kDa on SDS-PAGE (Figure 7A, lane 1). To ascertain the purified protein with protease activity, the crude and purified enzymes were further assayed with gelatin zymography. There was one clear hydrolytic band in the purified and crude enzyme on the gelatin zymography (Figure 7B, lanes 1 and 2), indicating no other proteins exerted PA beyond the 35 kDa protein band in crude enzyme. The purified enzyme obtained from DEAE Sepharose Fast Flow chromatography was further checked by hydrophilic interaction liquid chromatography. The purified enzyme displayed a major symmetrical peak with a retention time of 2.560 min in the HPLC, and the purity of the enzyme reached 98.8% ± 0.2% calculated from the target peak area to total peak area (Figure 7C).

The residual MCA were more than 90% in the presence of 0.02 mM pepstatin A (inhibitor of aspartate protease), 1 mM PMSF (inhibitor of serine protease), and 4 mM 2-iodoacetamide (inhibitor of cysteine protease), revealing that this enzyme did not belong to aspartate protease, serine protease, or cysteine protease. The enzyme activity was completely inhibited by 5 mM of ethylenediaminetetraacetic acid (EDTA), indicating that it was a metalloprotease. This result suggested the protease was different from other reported bacterial coagulants, such as those from *B. amyloliquefaciens* D4 [35], *B. amyloliquefaciens* JNU002 [17], *B. subtilis* YB-3 [27] and *B. licheniformis* USC13 [29], in both molecular weight and type, as listed in Table 1.

One of the essential characteristics of a milk-clotting protease suitable in the manufacture of cheese is to specifically cleave at or immediately adjacent to the Phe_105_–Met_106_ bond of κ-CN and release para-κ-casein and glycomacropeptide (GMP) [14]. κ-CN (5 mg·mL^−1^) was incubated with BD3526 protease at the final MCA of 0.1 SU mL^−1^ at 35 °C for 3 min, and the proteolytic peptides were determined by mass spectrometry analysis [51]. According to the detected peptides (Table 2), the cleavage sites were predominantly located in the macropeptide moiety of κ-CN at the Met_106_–Ala_107_, Glu_129_–Pro_130_, and Asp_148_–Ser_149_. Based on the occurrence frequency of each amino acid at the cleavage site, the Met_106_–Ala_107_ bond of κ-CN might be the primary proteolytic site of BD3526 protease and the other sites could be attributable to the consequent proteolysis of the hydrophilic peptide, κ-CN (f107–169).

**Table 2:** Identity of peptides produced from kappa-casein by BD3526 milk-clotting protease.

Observed Mass	Calculated Mass	Δppm	Missed Cleavages	Peptide Sequences	*n*
2464.3154	2464.3173	1.14	0	M.AIPPKKNQDKTEIPTINTIASGE.P	11
4394.2116	4394.2228	2.54	0	M.AIPPKKNQDKTEIPTINTIASGEPTSTPTTEAVESTVATLED.S	9

*n*, number of peptides.

### 2.7. Comparison of the BD3526 Protease with Commercial Coagulants

Apart from the yields of microbial proteases with MCA, MCA/PA ratio is also a very important criterion for evaluating their potential as rennet substitutes [35]. Milk-clotting protease with strong PA would excessively hydrolyze caseins, and thus led to cheese yield reduction, flavor and texture defects. PA of milk-clotting protease varies greatly in terms of determination and definition methods [32,52], which complicates the comparisons among different studies. Thus, MCA/PA of different milk-clotting proteases should be assessed under the same conditions, and similar to those employed in the cheese-making. Released amino acids (e.g., tyrosine) in whey, one of the assays usually employed to measure proteolysis, is efficient to reflect the PA of coagulant [47].

Curd forming and whey syneresis were positively correlated with the PA of coagulants. At 10-, 20-, 40-, 60- and 90-min intervals, curd forming and proteolysis by different coagulants including the BD3526 protease were assayed. Curds formed 10 min after the addition of coagulants remained intact, without excessive proteolysis and whey syneresis until 40 min. The increments of concentration of tyrosine in the whey resulted by Chy-MAX^®^, rennet, Marzyme^®^ 150 MG and BD3526 protease at 90 min were 0.02, 0.03, 0.09 and 0.12 μmol·mL^−1^, respectively (Table 3). The MCA/PA ratio of the *R. miehei* coagulant is the lowest among the commercially microbial coagulants tested [14,49,53], which was 1.6 to 4.5 folds higher than that of recombinant chymosin [44]. As the coagulants added at the ratio of 1/20 (*v*/*v*), the final MCA in the reconstructed skim milk was approximately 4 SU mL^−1^ and the MCA/PA ratios of chymosin, rennet, *R. miehei* coagulant and BD3526 protease were 200, 133.33, 44.44 and 33.33, respectively. The MCA/PA ratio of BD3526 protease was approximately the same level to that of *R. miehei* coagulant, which indicated the potential of BD3526 protease as a rennet substitute.

**Table 3:** Changes of tyrosine concentration in the whey of curded skim milk by different coagulants.

Coagulants	Tyrosine Concentration (µmol·mL^−1^)				
	10 min	20 min	40 min	60 min	90 min
calf rennet	0.87 ± 0.02	0.94 ± 0.03	0.92 ± 0.03	.90 ± 0.02	0.90 ± 0.03
recombinant chymosin (Chy-MAX^®^)	0.93 ± 0.03	0.91 ± 0.05	0.96 ± 0.06	0.96 ± 0.03	0.95 ± 0.04
*R.miehei* (Marzyme^®^ 150 MG)	0.95 ± 0.03	0.96 ± 0.04	0.96 ± 0.05	1.00 ± 0.04	1.04 ± 0.03
BD3526	0.90 ± 0.03	0.90 ± 0.04	0.96 ± 0.04	1.01 ± 0.04	1.02 ± 0.05

## 3. Experimental Section

### 3.1. Materials

Medium-heat sprayed skim milk powder (Fonterra Ltd., Auckland, New Zealand) used for skim milk agar preparation and MCA determination was composed of (%, *w*/*w*): fat 0.8, protein 33.4, lactose 54.1, mineral 7.9 and moisture 3.8. Wheat bran was purchased from a local market in Shanghai, and contained (%, *w*/*w*): protein 18.6, fat 6.2, carbohydrates 63.9, ash 3.38 and moisture 7.89. Marzyme^®^ 150 MG (*R. miehei*, 662 SU mg^−1^) and Chy-MAX^®^ (recombinant chymosin, *Aspergillusniger* var. *awamori*, 310 SU mg^−1^) were donated by DuPont Danisco (Vinay, France) and CHR. HANSEN A/S (Hørsholm, Denmark), respectively. Calf rennet (90 SU mg^−1^) was purchased from Al-amin Biotech Co., Ltd. (Shanghai, China). All other chemicals were obtained from commercial sources and were of analytical grade.

### 3.2. Strain Screening and Culture Conditions

Reconstituted skim milk (150 g·L^−1^) and agar solution (16.7 g·L^−1^) were prepared and sterilized at 118 °C for 15 min in an autoclave (Hirayama HVE-50, Tokyo, Japan), respectively. The sterilized reconstituted skim milk and agar solution were aseptically mixed in a ratio of 1:9 (*v*/*v*) and then poured plates. Raw yak milk samples collected in Tibet, southwestern China were serially diluted with sterile physiological saline and spread on skim milk agar plates. The plates were then incubated at 30 °C for 48 h. A colony, designated as BD3526, with an obvious casein hydrolysis circle surrounded with a casein precipitation zone, was selected and further purified by streaking on skim milk agar plates. The strain was deposited in the China General Microbiological Culture Collection Center (CGMCC No. 8333) as well as German collection of microorganisms and cell cultures (DSM No. 28815). The strain BD3526 was routinely propagated on Tryptone Yeast Cystine agar (TYC), which contained (g·L^−1^) tryptone 15, yeast extract 5, l-cystine 0.2, sucrose 50, sodium acetate 20, sodium hydrogen carbonate 2, disodium hydrogen phosphate 2, sodium chloride 1, sodium sulfite 0.1, agar 15, and the pH value was adjusted to 7.2. Stock culture was maintained at −80 °C in sterile 10% (*w*/*v*) skim milk. Prior to the experiments, the strain was activated twice on TYC agar.

### 3.3. Morphological Observations and Characterization

Freshly cultured BD3526 cells on nutrient agar (Merck, Darmstadt, Germany) slant at 30 °C for 24 h were employed for morphological observation. After Gram staining, the slide with BD3526 was immediately visualized under ×1500 magnification using an optical microscope (Olympus BX-60, Tokyo, Japan). A loop of fresh culture of BD3526 was suspended in 5 mL of sterilized phosphate buffered saline (PBS), and aliquots of the suspension was checked for cell motility under phase contrast microscope (Olympus BX-60, Tokyo, Japan). The morphology of the cells was observed using a scanning electron microscope (SEM) (FEI Quanta 200, Eindhoven, Netherlands) and the existence of flagella was observed with an atomic force microscope (AFM) (Nanonavi E-Sweep, SII Nano Technology, Tokyo, Japan).

### 3.4. Preparation of Inoculums

A single colony of BD3526 on TYC agar was inoculated into 20 mL sterilized TYC broth in a 100 mL Erlenmeyer flask, and cultivated at 30 °C on a rotary shaker at 180 rpm for 18–20 h. The cultivated TYC broth, with viable cell counts approximately to 5.0 × 10^8^ CFU mL^−1^, was employed as inoculum. Enumeration of viable cell counts was carried out after the sample was serially 10-fold diluted with sterilized PBS, and aliquots of the diluted sample was spread on TYC agar, cultivated aerobically at 30 °C for 48 h.

### 3.5. Wheat Bran Broth and Cultivation Conditions

The optimal wheat bran concentration for BD3526 to express protease with MCA were determined by adding varied wheat bran mass (0.5, 1.0, 1.5, 2.0, 2.5 and 3.0 g) to 50 mL deionized water in 250 mL Erlenmeyer flasks and then sterilized at 121 °C for 20 min. The sterilized wheat bran broth was seeded with the inoculum (~5.0 × 10^8^ CFU mL^−1^) at a ratio of 3% (*v*/*v*) and incubated on a rotary shaker at 30 °C at 180 rpm for 48 h. Samples at different intervals were taken out and centrifuged at 5000× *g* for 5 min to remove insoluble materials. The supernatants were diluted with 20 mM phosphate buffer (PB) (pH 6.0) for MCA assay.

The optimal rotation speed was determined using 3% (*w*/*v*) wheat bran broth, 50 mL in a 250 mL Erlenmeyer flask, 3% (*v*/*v*) inoculum, at 30 °C and different rotation speeds from 140 to 300 rpm for 24–32 h. The optimal volume of liquid was determined using 3% (*w*/*v*) wheat bran broth, 3% (*v*/*v*) inoculum, at 30 °C and 300 rpm from 10 mL to 50 mL in a 250 mL Erlenmeyer flask for 20–32 h. The optimal cultivation temperature was determined using 3% (*w*/*v*) wheat bran broth, 3% (*v*/*v*) inoculum, 30 mL in a 250 mL Erlenmeyer flask, at 300 rpm from 20 to 42 °C for 20 h.

### 3.6. Enzyme Preparation and Purification

The supernatant of cultivated broth with 3% (*v*/*v*) wheat bran at 30 °C, 180 rpm for 30 h was collected and the protease with MCA was precipitated by ammonium sulfate at 60% saturation. The precipitate was lyophilized and defined as the crude enzyme. The crude enzyme was redissolved in 20 mM PB (pH 7.0) at concentration of (0.5 g·mL^−1^) and loaded on a DEAE Sepharose Fast Flow (GE Healthcare Bio-Sciences AB, Uppsala, Sweden) column (ID 3.6 cm × 30 cm) previously equilibrated with 20 mM PB (pH 7.0). Elution was carried out with a linear gradient of NaCl from 0 to 0.75 M at a flow rate of 5 mL·min^−1^ in 20 mM PB (pH 7.0). The elution was monitored at 280 nm and 10 mL fractions were collected. Fractions with MCA were pooled, lyophilized and defined as the purified enzyme.

### 3.7. Gel Electrophoresis

Ten microliter of crude and purified enzymes solutions were assessed by SDS-PAGE using 12% (*w*/*v*) acrylamide. Molecular weight marker proteins MP102 (Tiangen Biotech Co., Ltd., Beijing, China) were used as standards. To determine the band responsible for the protease activity, gelatin zymography was carried out according to the method described by Vishwanatha with a slight adjustment [54]. Gelatin zymography was carried out on 12% acrylamide gels with 0.1% (*w*/*v*) gelatin in the separating gel. Precision Plus Protein™ All Blue Standards (Bio-Rad, Hercules, CA, USA) were used as molecular weight markers for gelatin zymography. Electrophoresis was carried out at 4 °C at a constant voltage of 110 V for 90 min when the tracking dye (bromophenol blue) exited the gel. After electrophoresis, the gel was washed three times with 1.5% (*w*/*v*) Tween 80 solution to remove SDS and recover enzyme activity, and incubated in the 20 mM Tris-HCl (pH 7.0) at 30 °C for 24 h. The gel was further stained with Coomassie brilliant blue R-250 for 30 min and then decolored to see the apparent hydrolytic band.

### 3.8. Hydrophilic Interaction Liquid Chromatography

The purified enzyme was further checked by hydrophilic interaction liquid chromatography (HILIC). The analysis was performed on an Agilent1260 Infinity system (Agilent Technologies, Palo Alto, CA, USA), equipped with a SeQuant^®^ ZIC^®^-HILIC (Merck) column (ID 4.6 × 250 mm, 5 µm, and 200 Å). Ten microliter of the purified enzyme solution of 0.5 mg·mL^−1^ was loaded, and eluted with a mixture of acetonitrile/20 mM ammonium acetate solution (80:20, *v*/*v*) as the mobile phase at a flow rate of 1.0 mL·min^−1^. The eluent was monitored with a diode array detector (DAD) (Agilent, G1315D) at 280 nm. All samples were filtered through a 0.22 μm membrane before injection.

### 3.9. Determination of Milk-clotting Activity and Proteolytic Activity

MCA was determined using the method described by Arima [55]. Reconstituted skim milk (100 g·L^−1^) was freshly prepared, supplemented with 10 mM calcium chloride and stored overnight at 4 °C for complete hydration. The pH value of the milk was adjusted to 6.0 with 1 M HCl before use. A test tube containing 10 mL skim milk was pre-incubated at 35 °C for 10 min and then 0.5 mL diluted enzyme solution was added. All enzyme samples were diluted with 20 mM, pH6.0 PB, and coagulation time of the diluted enzyme was adjusted to between 1 and 3 min. The mixture was thoroughly vortexed and the time from the addition of the enzyme to the formation of the first visible clot was recorded. MCA was calculated with the following formula [35]:

(1) SU = (2400 × 10 × D)/0.5T

where *T* is the milk-clotting time (s), and *D* is the dilution of the enzyme. One Soxhlet unit (SU) of milk-clotting activity is defined as the amount of enzyme required to clot 1 mL of substrate within 40 min at 35 °C.

PA was determined according to the casein digestion method described by Shieh [28]. In brief, 2.5 mL 1.2% (*w*/*v*) of casein solution in 20 mM PB (pH 6.0) was added 0.5 mL enzyme solution, and the mixture was incubated at 35 °C for 10 min. After incubation, 2.5 mL (0.44 M) trichloroacetic acid (TCA) was added to quench the reaction, followed by centrifugation to remove sediments. One milliliter of the supernatant was added with 2.5 mL NaOH (0.28 M) solution and 0.75 mL phenol reagent (Folin-Ciocalteu phenol solution/water = 1:1). After the mixture was kept at 35 °C for 15 min, optical density (OD) at 660 nm was measured with a Specord 205 spectrophotometer (Analytik Jena AG, Jena, Germany). A calibration curve of tyrosine content was constructed by referring to absorbance at 660 nm obtained with standards containing 0, 20, 40, 60, 80 and 100 μg of tyrosine.

### 3.10. Evaluation of Protein Hydrolysis of the BD3526 Coagulant

Solutions of calf rennet, Chy-MAX^®^, Marzyme^®^ 150 MG and the BD3526 coagulant were prepared and adjusted to 160 SU mL^−1^. Milk curds clotted by different enzymes were observed and assayed at intervals of 10, 20, 40, 60 and 90 min. After centrifugation at 10,000× *g* for 5 min, whey was separated from the curds. The degree of protein hydrolysis was determined and expressed by increment of tyrosine concentration in whey.

### 3.11. Statistical Analysis

All of the data were expressed as means ± standard deviation (SD) from at least three replicates. One-way and two-factor analysis of variance (ANOVA) were performed. One-way ANOVA was used to compare the mean levels of various cultivation temperature and MCA. In evaluating the combined effects of wheat bran concentration, rotation speed, liquid volume and cultivation time on MCA, a two-factor ANOVA was used. All statistical analyses were performed using the Statistical Analysis Software (SAS 9.2, SAS Institute Inc., Cary, NC, USA). The confidence level was set at *p* < 0.05 for statistical significance. 

## 4. Conclusions

*Paenibacillus* spp. BD3526, a strain newly isolated from raw yak milk sample expressed protease with high MCA in wheat bran broth. Higher availability of dissolved oxygen favored a high cell density of BD3526 and ultimately resulted in a high expression of MCA. The enzyme was a 35 kDa metalloprotease and quite different from the reported bacterial coagulants in both molecular weight and type, implying that it might be a novel enzyme. The milk-clotting mechanism of the enzyme was the cleavage of the Met_106_–Ala_107_ bond in κ-CN. No excessive proteolysis during the procedure of milk-clotting indicates the BD3526 enzyme could be served as a potential microbial coagulant. Further research in enzyme characterization and application is thus necessary.
